# Notch3-Dependent Effects on Adult Neurogenesis and Hippocampus-Dependent Learning in a Modified Transgenic Model of CADASIL

**DOI:** 10.3389/fnagi.2021.617733

**Published:** 2021-05-21

**Authors:** Fanny Ehret, Ricardo Moreno Traspas, Marie-Theres Neumuth, Bianca Hamann, Daniela Lasse, Gerd Kempermann

**Affiliations:** ^1^German Center for Neurodegenerative Diseases (DZNE), Dresden, Germany; ^2^Center for Regenerative Therapies Dresden, TU Dresden, Dresden, Germany

**Keywords:** adult neurogenesis, CADASIL, dentate gyrus, hippocampus, Notch3, spatial learning, human brain, mouse model

## Abstract

We and others have reported that Notch3 is a regulator of adult hippocampal neurogenesis. *Cerebral Autosomal Dominant Arteriopathy with Subcortical Infarcts and Leukoencephalopathy* (CADASIL), the most common genetic form of vascular dementia, is caused by mutations in *Notch3*. The present study intended to investigate whether there is a correlation between altered adult hippocampal neurogenesis and spatial memory performance in CADASIL transgenic mice. To overcome visual disabilities that hampered behavioral testing of the original mice (on an FVB background) we back-crossed the existing TgN3^*R169C*^ CADASIL mouse model onto the C57BL/6J background. These animals showed an age-dependent increase in the pathognomonic granular osmiophilic material (GOM) deposition in the hippocampus. Analysis in the Morris water maze task at an age of 6 and 12 months revealed deficits in re-learning and perseverance in the CADASIL transgenic mice. Overexpression of Notch3 alone resulted in deficits in the use of spatial strategies and diminished adult neurogenesis in both age groups. The additional CADASIL mutation compensated the effect on strategy usage but not on adult neurogenesis. In brain bank tissue samples from deceased CADASIL patients we found signs of new neurons, as assessed by calretinin immunohistochemistry, but no conclusive quantification was possible. In summary, while our study confirmed the role of Notch3 in adult neurogenesis, we found a specific effect of the CADASIL mutation only on the reversion of the Notch3 effect on behavior, particularly visible at 6 months of age, consistent with a loss of function. The mutation did not revert the Notch3-dependent changes in adult neurogenesis or otherwise affected adult neurogenesis in this model.

## Introduction

Cerebral Autosomal Dominant Arteriopathy with Subcortical Infarcts and Leukoencephalopathy (CADASIL), the most common genetic cause of stroke and vascular dementia, results in accumulation of granular osmiophilic material (GOM) in small and medium sized arteries (Chabriat et al., 2009). We previously identified GOMs in the vasculature of the adult hippocampus in an established transgenic mouse model overexpressing *Notch3* (N3) with a CADASIL-causing point mutation (Ehret et al., 2015). In that model we saw effects on adult hippocampal neurogenesis but could not assess any potential role in hippocampus-dependent behavior, because the existing mice, which are on a FVB background, cannot be feasibly tested for spatial memory because of a genetic visual deficit (Voikar et al., 2001; Farley et al., 2011). The current project aimed at overcoming that limitation in order to support our original hypothesis that neurogenesis-related impairments due to the N3 mutation in neural stem cells might contribute to the overall phenotype in CADASIL.

The hippocampus is one of the first structures affected in dementias, playing an essential role in memory processing. Particularly the consolidation of declarative memory, which includes semantic and episodic memory, is processed by the hippocampus (O’Keefe and Dostrovsky, 1971; reviewed by Morris, 2007). Even though only a few studies have specified the memory deficits seen in CADASIL patients and despite the limitations of the neuropsychological assessment strategies used, some interesting commonalties have arisen. CADASIL patients often present deficits in episodic memory, executive function and working memory prior to stroke and age-dependent cognitive decline (Buffon et al., 2006; Epelbaum et al., 2011). This pattern of memory impairment suggests, besides the involvement of subcortical-frontal regions, connections to the hippocampal subfields. Furthermore, a correlation has been identified between hippocampal volume and cognitive performance in CADASIL patients, independent of vascular lesions (O’Sullivan et al., 2009). Hence, these findings point to potential role of the hippocampus in the manifestation of CADASIL.

Besides its pivotal role in memory processing, the hippocampus is also unique in that it maintains lifelong adult neurogenesis. The current view suggests that neurogenesis in this region provides increased plasticity, which is critical for lifelong cognitive flexibility (Burghardt et al., 2012; Kempermann, 2012; Spalding et al., 2013; Garthe et al., 2014). This process is tightly controlled by internal and external factors acting on residing stem and progenitor cells. The Notch super-family is one of such transcriptional regulators that play a central role in stem cell quiescence and maintenance, progenitor cell proliferation and differentiation in the developing and adult brain (Ehm et al., 2010; Imayoshi et al., 2010; Lugert et al., 2010). Since CADASIL is due to a diversity of point mutations in N3, the Notch signaling pathway is of particular relevance. Although most of the work focuses on *Notch1* (N1), some aspects regarding the critical function of N3 in stem cell quiescence and neurogenesis have been identified (Kawai et al., 2017; Than-Trong et al., 2018). In adult mice, N3 is expressed in neural stem and progenitor cells in the subependymal zone of the lateral ventricles (Kawai et al., 2017) and the subgranular zone of the hippocampus (Ehret et al., 2015). Importantly, our previous research identified N3 as a critical regulator of precursor cell proliferation and differentiation in the neurogenic niche of the murine hippocampus (Ehret et al., 2015). Although a detailed analysis in human cells and tissues is still lacking, these findings already suggest that N3 might exert regulatory influences on neuronal plasticity that could impact hippocampus-dependent learning and memory.

The N3 signaling pathway is of outstanding relevance in CADASIL, since mutations in the N3 gene are disease-causative. We already proved the presence of altered hippocampal neurogenesis independent to overt vascular abnormalities in a transgenic mouse model of CADASIL (Ehret et al., 2015). In the current study we aimed at confirming whether N3 dysfunction might be an additional underlying mechanism accounting for the learning and memory deficits seen in CADASIL patients, possibly independent of vascular dysfunction but related to a dysfunction in neural stem cells in the hippocampus. For that purpose, we backcrossed the established mouse model TgN3^*R169C*^ (Joutel et al., 2010) onto a C57BL/6J background in order to allow unconstrained behavioral testing in the classical Morris water maze task. We used a concrete testing paradigm, which we had previously identified as highly sensitive to the contribution of adult-generated neurons to spatial learning (Garthe et al., 2009, 2014). Additionally, we intended to investigate whether we could find signs of altered adult hippocampal neurogenesis in samples from CADASIL patients.

## Materials and Methods

### Animals and Tissue Preparation

TgN3^WT^ and TgN3^*R169C*^ generated by Joutel et al. (2010) and crossed back for 10 generations to C57BL/6J (Charles River), were maintained at the CRTD – Center for Regenerative Therapies Dresden, Dresden, Germany. WT littermates from both strains were used as controls. All experiments were conducted in accordance with the applicable European and National regulations (Tierschutzgesetz) and approved by the responsible authority (Landesdirektion Dresden, approval number 24-9168.11-1/2012-26). Animals were maintained on a 12 h light/dark cycle with food and water provided *ad libitum.* TgN3^WT^ and TgN3^*R169C*^ mice were genotyped by PCR (*Notch3* forward: 5′ TTC AGTGGTGGCGGGCGTC 3′ *Notch3* reverse: 5′GCCTACAGGTGCCACCATTA CGGC 3′; Vector forward: 5′ AACAGGAAGAATCGCAACGTTAAT 3′ Vector reverse: 5′ AATGCA GCGATCAACGCCTTCTC 3′). *Notch3* PCR products from TgN3^WT^ and TgN3^*R169C*^ mice were sequenced and the genetic mutation site was identified to confirm the correct genotype of the strains used. The effect on protein expression has been analyzed in the original TgN3^WT^ and TgN3^*R169C*^ mice and is shown in Supplementary Figure 4.

Female and male C57BL/6J, TgN3^WT^, and TgN3^*R169C*^ mice received intraperitoneal injections of 5-bromo-2-deoxyuridine (BrdU, Sigma-Aldrich) at 50 mg/kg, dissolved in sterile 0.9% NaCl (10 mg/ml). BrdU was delivered three times every 6 h for 6 months old animals and seven times every 24 h for 12 months old animals. Mice were killed 28 days after BrdU administration by using a mixture of ketamine and xylazine and transcardial perfusion with 0.9% NaCl and 4% paraformaldehyde (PFA). The brains were left in 4% PFA for 24 h at 4°C, transferred to 30% sucrose and cut into 40 μm thick serial coronal sections on a freezing microtome (HM430, Thermo Scientific). Sections were stored at −20°C in cryoprotectant solution (25% ethylene glycol, 25% glycerol in 0.1 M phosphate buffer, pH 7.4).

### Human Samples

Post-mortem human hippocampus samples (*N* = 7 CADASIL and *N* = 6 Control samples) were obtained from the CADASIL brain bank, Leiden University, Netherlands (age range 48–70 years, including both sexes). Out of the seven CADASIL patients, six had a mutation on exon 4 and one in exon 8. The modeled R169C mutation was not present in the human samples. All participants gave informed consent that their samples could be used for research and publication, nevertheless to prevent traceability the data are only presented in charts. For details on the influence of age, sex or post mortem delay on the obtained (but ultimately inconclusive) data see Supplementary Figure 1.

### Immunohistochemistry

#### Animal Tissue

As described previously (Kempermann et al., 2003), for BrdU detection every sixth brain section from C57BL/6J, TgN3^WT^, TgN3^*R169C*^ animals at 6 months and 12 months of age were used (group size at 6 months WT = 15, TgN3 both 12 and at 12 months WT = 19, TgN3 both 13). Briefly for BrdU detection, sections were quenched with 0.6% H_2_O_2_ for 30 min, incubated in 2 N HCl for 30 min at 37°C followed by 1 h blocking with TBS^++^ (10% donkey serum, 0.2% Triton-X 100 in TBS) and incubation in monoclonal rat anti-BrdU antibody (1:500; OBT0030, Serotec) overnight at 4°C in TBS^+^ (3% donkey serum in TBS). For calretinin detection an analog protocol was used, but without the HCl denaturing step. Sections were incubated with primary antibody rabbit anti-calretinin (1:2000, CR7697, Swant). To determine the total number of labeled cells, the peroxidase method was used with biotinylated donkey anti-rat or anti-rabbit antibody (similarly as described for the human tissue). However, no counter stain was performed and sections were mounted on gelatine-coated slides and cover-slipped with Neo-Mount (Merck). For phenotyping of BrdU^+^ cells, immunofluorescence was performed. Free-floating sections were washed, incubated with 2 N HCL for 30 min at 37°C, blocked for 1 h in TBS^++^ and incubated over night at 4°C with the following primary antibodies: rat anti-BrdU (as above), mouse anti-NeuN (1:500; MAB377, Millipore), rabbit anti-S100β (1:1000; ab52642, Abcam). The next day, sections were washed and incubated with secondary antibody for 2 h at RT. All secondary antibodies were purchased from Dianova: anti-mouse Dye-Light 549, anti-rat Alexa 488, anti-rabbit Alexa 647. After a final washing step, sections were incubated for 10 min in Hoechst (1:4000 in TBS; 33342, Sigma-Aldrich) and then mounted on glass slides using 2.5% PVA-DABCO.

#### Human Tissue

Paraffin-fixed sections of 5 μm thickness were taken from three different blocks per human hippocampus. Per patient six sections two per each block were analyzed. Tissue sections were dewaxed with xylene and rehydrated with decreasing concentrations of ethanol. Unmasking was performed using 1 mM EDTA (pH 8) at 110°C in a pressure cooker for 5 min. Sections were afterward incubated with 3% H_2_O_2_ for 30 min, blocked for 1 h in TBS^+^ (containing 10% donkey serum) and subsequently Avidin-Biotin blocked (SP2001, Vector labs) followed by incubation with calretinin antibody (1:2000, CR7697, Swant) overnight at 4°C in TBS^+^ (containing 5% donkey serum).

To determine the number of labeled cells, the peroxidase method (Vectastain Elite ABC kit, Vector laboratories) was used with biotinylated donkey anti-rabbit antibody (1:500, Dianova) and diaminobenzidine (DAB, Sigma) as a chromogen. Sections were finally haematoxylin counter stained (Richard-Allan Scientific Hematoxylin, Thermo Scientific), sequentially dehydrated and cover-slipped with Entellan (Sigma-Aldrich).

#### Image Quantification

Bromodeoxyuridine and calretinin cell quantification were performed on coded slides using widefield light microscopy with a Leica microscope (DM 750, Leica). The number of immunopositive (+) cells present in the SGZ of the DG was quantified by manually counting all DAB-positive cells present within 40 μm of the granular cell layer of the DG formation (i.e., from Bregma −1.06 to Bregma −3.88). Fluorescence images for BrdU phenotyping were obtained on a fluorescence microscope with 2D structured illumination (ApoTome.2, Zeiss). Images were processed with Zen software (Zeiss), Fiji and Illustrator CS5 (Adobe). Only general contrast and color level adjustments were made; otherwise images were not digitally manipulated. Net neurogenesis and gliogenesis were calculated for each animal separately by multiplying the proportion of BrdU differentiation obtained through fluorescent double labeling (NeuN^+^BrdU^+^ or S100β^+^BrdU^+^) with the number of BrdU^+^ cells obtained through DAB.

### Morris Water Maze Task

Mice were trained in the reference memory version of the Morris water maze task to locate a hidden escape platform in a circular pool (2 m diameter). In opaque water (by adding non-toxic titan white) at 19–20°C, mice had to swim six trials a day for five consecutive days for 6 months-old mice, or seven consecutive days for 12 months-old mice. The experimental design (see Figures 2A, 4A) contained 3 or 4 days acquisition (for 6 and 12 months old mice, respectively) followed by 2 or 3 days of reversal, where the platform position was switched to the opposite quadrant. Mice were released every day from a new starting position and allowed to search for the hidden platform for a maximum of 120 s. During each day the starting position remained constant. At the end of each trial and irrespective of trial performance, mice were guided to the platform and allowed to remain there for at least 15 s. At the last day a final trial with a visual platform in a new position was performed to evaluate the visual abilities of the mice. Swim paths were recorded using EthoVision (Noldus) and further analyzed using Matlab (The Mathworks). To visualize spatial preference for the goal position in the probe trials, a heat map like occupancy plot was divided into 10 cm × 10 cm wide sectors and the probability of an animal to be found in each sector was calculated. Search strategies were classified according to parameters and an algorithm described in our previous study (Garthe et al., 2014), originally based on (Balschun et al., 2003). Strategies were defined by no more than two parameters that are not dependent on pool dimensions. In detail, the swim path data from EthoVision (Noldus) were used to derive the time-tagged x, y-coordinates for the dominant search strategies by an algorithm implemented in Matlab (Mathworks). For analysis of these search strategies a logistic regression model was applied and evaluated using the working environment R (for details see statistics).

### Electron Microscopy

Female and male mice were perfused with 0.9% NaCl followed by Karnovsky’s fixative (2% glutaraldehyde, 2% paraformaldehyde, 20 mM HEPES). The brains were left in Karnovsky solution overnight followed by vibratome sectioning (Model 1200 Leica) at a thickness of 200 μm. Tissue was contrasted for 2 h in 1% OsO_4_ at 4°C, washed, *en bloc* contrasted with 1% uranyl acetate for 2 h at 4°C, washed and gradually dehydrated in ethanol. This was followed by infiltration of Epon 812 resin, embedding in molds and polymerization at 60°C for 24 h. Semithin sections were stained with 1% toluidine blue and 0.5% Borax (TB/Borax) and inspected with widefield microscope BZ 8000 (Keyence). Ultrathin 70 nm sections were analyzed on a transmission electron microscope (Morgagni 268D, FEI).

### Statistics

For analysis of human post-mortem samples we used unpaired *t*-test since data was normally distributed.

For analysis of adult neurogenesis rates in transgenic animals we used one-way ANOVA, since the data passed the D’Agostino & Pearson normality test, with Bonferroni *post-hoc* test.

For statistical analysis of the effect of WT, TgN3^WT^, and TgN3^*R169C*^ on water maze performance (swim speed, latency, and path length), we performed the non-parametric testing of Friedman with Dunn’s *post-hoc* test since the data was not normally distributed. For probe trial performance we used Kruskal–Wallis test at 6 months of age since data was not normally distributed, and one-way ANOVA at 12 months of age since data was normally distributed (tested with D’Agostino & Pearson normality test).

For statistical analysis of the effect of WT, TgN3^WT^, and TgN3^*R169C*^ on the search strategies, we applied a logistic regression (LR) (as discribed previously Garthe et al., 2016). Using the LR model with nested effect, we estimated the odds ratios for the three different genotypes (WT, TgN3^WT^, and TGN3^*R169C*^), thus comparing the chance of using more versus less hippocampus-dependent search strategies. Corresponding *p*-values are based on ANOVA. Calculations for search strategy analysis were done in working environment R, all other statistical calculations were done in Prism 7 (GraphPad).

## Results

Based on the previous observation of reduced adult hippocampal neurogenesis (Ehret et al., 2015) in the CADASIL transgenic mouse model established by Joutel et al. (2010), we aimed at identifying behavioral deficits in hippocampus-dependent spatial learning by the Morris water maze task. Therefore, this CADASIL transgenic mouse model was back-crossed onto the C57BL/6J background (B6) to overcome the limitations of the FVB/N background (Pugh et al., 2004) like impaired vision (Voikar et al., 2001).

### Accumulation of Granular Osmiophilic Material Starts at 6 Months in CADASIL Transgenic Mice on the B6 Background

**FIGURE 1:**
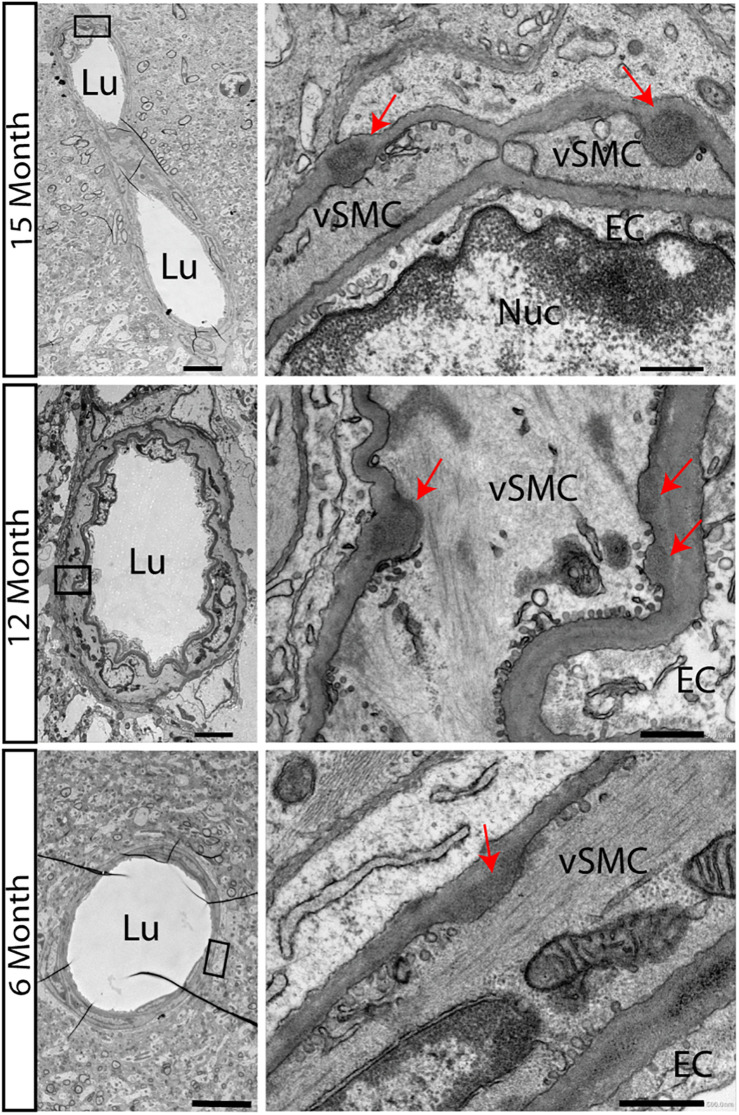
Accumulations of GOMs in hippocampus of TgN3^*R169C*^ mice. Electron microscopic analysis of the hippocampus in TgN3^*R169C*^ mice showed accumulation of electron dense granular osmiophilic material at 15 and 12 months of age and less dense accumulation already at 6 months of age, as highlighted by the red arrow. Scale bar for left column 5 μm, right column 0.5 μm. EC, endothelial cells; Lu, Lumen; Nuc, Nucleus; vSMC, vascular smooth muscle cells.

We first confirmed that in the new backcrossed model, neuropathological characteristics of CADASIL would be maintained. Accumulations of electron dense GOMs in small and medium sized arteries, a key feature of the disease process, appears in some transgenic animal models but not in all. Especially in the used animal model of TgN3^*R169C*^ on FVB/N background, GOM deposits started to occur at 5 months in the pial arteries and became widely distributed throughout the brain by 10–12 months of age (Joutel et al., 2010). On the B6 background, we now carried out a detailed electron microscopic analysis to observe the disease progression. Surprisingly, we found GOM accumulations already at 6 months of age in TgN3^*R169C*^ on C57BL/6 background in the hippocampus (Figure 1). GOMs can be found close to the 3rd ventricle and around medium-sized vessels of the hippocampal fissure.

### Deficits in Hippocampus-Dependent Learning in Aged TgN3^WT^ and TgN3^*R169C*^ Mice on the B6 Background

**FIGURE 2:**
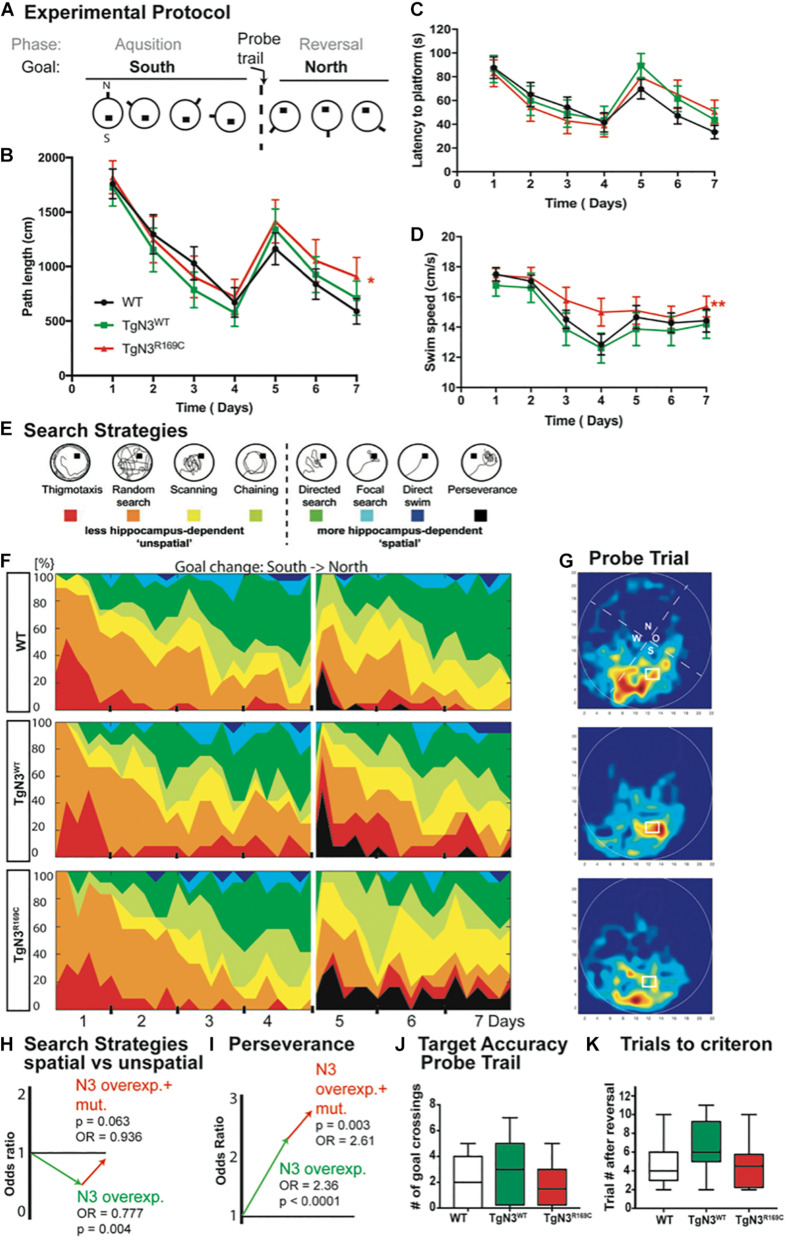
Impaired spatial learning in the Morris water maze task in *Notch*3 and CADASIL transgenic mice at 12 months of age. **(A)** The experimental protocol of the Morris water maze task adjusted for aged animals and thus containing 4 days of acquisition and 3 days of reversal. **(B)** Analysis of path length showed an impaired performance after platform relocation in CADASIL mice (* represents *p* < 0.05). **(C)** Analysis of latency shows similar trends but no significant effect since the swim speed is significant higher in TgN3^*R169C*^. **(D)** Swim speed of CADASIL mice differed significantly compared to N3 transgenic controls (**p < 0.01). **(E)** Examples for classification of search strategies used to define “unspatial” vs. “spatial” more hippocampus-dependent patterns. **(F)** Contribution of the respective search strategies to group performance, color code as indicated in **(E)**. **(G)** Heat maps from the respective animal groups of the probe trial performance (only the first 30 s). **(H)** Graphic illustration of the induced change in the odds to choose a more hippocampus-dependent strategy as uncovered by logistic regression analysis based on previous classification shown in **(E)**. **(I)** Graphic illustration of the induced change in the odds for perseverance based strategy in the reversal phase of the Morris water maze task. **(J)** Analysis of former goal crossings as an indicator of target accuracy in the probe trials. No impairment in previous goal location can be seen in transgenic mice. **(K)** Number of trials an individual animal needed to regain the average path length of day 4.

An extended protocol was used for this task, consisting of 4 days of acquisition and 3 days of reversal, to cope with age-dependent deficits (experimental protocol Figure 2A). At 12 months of age, the transgenic mice showed specific deficits in relearning of a new platform position in the old context (reversal) while the initial acquisition was not impaired. In detail, the analysis of latency to find the hidden platform was unimpaired (Chi-Square = 0.857, *p* = 0.768; Figure 2C), but since swim speed differed significantly (Chi-Square = 12.29, *p* = 0.003 with TgN3^WT^ < TgN3^*R169C*^, *p* = 0.0015; Figure 2D), the analysis of path lengths is a more reliable measure (Chi-Square = 6, *p* = 0.05 with TgN3^WT^ vs. TgN3^*R169C*^ *p* = 0.048; Figure 2B). Relearning of a new platform position was impaired in CADASIL mice, as the analysis of path-length during reversal showed (Chi-Square = 6, *p* = 0.027 with WT vs. TgN3^*R169C*^ *p* = 0.04). Nevertheless, the initial memory of the previous platform position was not impaired, as analyzed during a probe trial by measuring the time the mouse spent in the relevant target zone [*F*_(2,40)_ = 1.667, *p* = 0.20] and the number of former goal crossings [*F*_(2,40)_ = 0.86 *p* = 0.43; Figures 2G,J]. In addition, we asked how many trials it took each mouse after goal reversal to regain a mean path length that was equal or shorter than on the last day of acquisition. Although there were variations between the different lines, no statistically significant difference could be detected [ANOVA *F*_(2,40)_ = 2.45, *p* = 0.09; Figure 2K]. The low *p*-value of 0.09 nevertheless indicates that further studies with increased power might reveal an effect of the mutation on the N3-dependent impairment.

In conclusion, the old platform position could be easily remembered, but CADASIL TgN3^*R169C*^ mice showed deficits in up-dating the old context. A detailed analysis of the search strategies revealed that the mice used different strategies to locate the hidden platform. These strategies are indicative of the underlying cognitive processes and brain structures involved (Balschun et al., 2003; Garthe et al., 2009). Thus, we analyzed the search patterns (Figure 2E) of the mice while they were navigating to the hidden goal during task acquisition (days 1–4) and the reversal (days 5–7). Specifically, we compared the ratio of spatially directed effective search patterns to less directed patterns and thus less effective strategies shown by the TgN3^WT^ and TgN3^*R169C*^ mice compared to WT mice (Figures 2F,H). Using a logistic regression, we statistically assessed changes in the chance (odds) for using either a more or less hippocampus-dependent strategy. The estimated odds-ratio (OR) for transgenic mice overexpressing N3 compared to WT was OR = 0.777 (*p* = 0.004), whereas the CADASIL mutation resulted in estimated OR = 0.936 (*p* = 0.063). Consequently, overexpression of N3 significantly impaired the use of spatial, more hippocampus-dependent strategies, while the CADASIL mutation *per se* (in comparison to N3 overexpression) resulted in less severe deficits in the use of spatial strategies. In the reversal phase, perseverance could be seen predominantly in the N3 and CADASIL transgenic mice. We therefore applied the logistic regression model to the perseverance and found a significantly increased perseverance in CADASIL mutant mice (OR = 2.61, *p* = 0.003) in addition to the perseverance seen in N3 overexpressing mice (OR = 2.36, *p* < 0.001; Figure 2I). These findings highlight that the R169C mutation resulted in deficits in up-dating of the allocentric map at this age, thereby resulting in an increased perseverance. These deficits are potentially due to a combined effect of vascular changes in the hippocampal niche (Ehret et al., 2015) and reduced adult neurogenesis. To confirm these neurogenic deficits in the modified mouse model, we carried out histological analysis of adult hippocampal neurogenesis.

### Deficits in Adult Neurogenesis in Aged N3 and CADASIL Transgenic Mice on the B6 Background

We had previously found alterations in adult neurogenesis in these N3 and CADASIL transgenic mouse models on the FVB/N background (Ehret et al., 2015). In the new modified transgenic mouse model deficits due to N3 overexpression could be found at 12 months of age, which is in agreement with our previous observation. Also in line with the previous report, the CADASIL mutation did not additionally impact precursor proliferation and neuronal survival, as only differences to WT animals could be detected but not to the N3 overexpressing controls in this age group.

**FIGURE 3:**
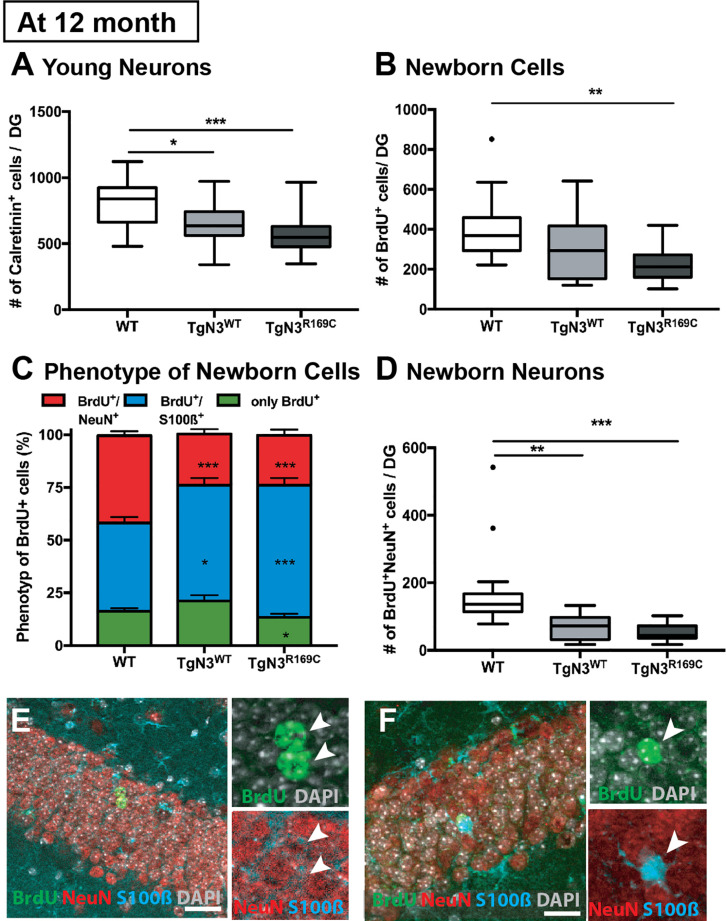
Reduced adult neurogenesis in *Notch3* and CADASIL transgenic mice at 12 months of age. **(A)** The number of calretinin^+^ cells, as a marker for young neurons, is reduced in the SGZ of the DG of Notch3 and CADASIL transgenic mice. **(B)** Analysis of adult neurogenesis by the number of BrdU^+^ cells 28 days post injection showed a reduction in CADASIL transgenic animals. **(C)** Phenotyping of BrdU^+^ cells located in the SGZ of the DG with neuronal marker (NeuN) and astrocyte marker (S100β) showed decreased neuronal differentiation and an increased astrocytic differentiation of Notch3 and CADASIL transgenic mice compared to WT, Data = Mean ± SEM. **(D)** Net neurogenesis, as calculated by number of BrdU^+^/NeuN^+^ cells, is reduced in Notch3 and CADASIL transgenic mice. **(E,F)** Representative image from TgN3^*R169C*^ mice of two BrdU^+^/NeuN^+^ neurons **(E)** and a BrdU^+^/S100β^+^ astrocyte **(F)**, scale bar 30 μm. *N* = 19 for WT and 13 for TgN3^WT^ and TGN3^*C169C*^ for all graphs. Asterisks indicate statistical significance (*p < 0.05, **p < 0.01, ***p < 0.005); DG, dentate gyrus; SGZ, subgranular zone.

In detail, analysis of adult neurogenesis by BrdU revealed a reduction in cell survival in TgN3^*R169C*^ [ANOVA *F*_(2,44)_ = 5.54, *p* = 0.007, with TgN3^*R169C*^< WT *p* = 0.006; Figure 3B]. Further phenotyping of BrdU^+^ cells showed a decreased proportion of neuronal differentiation [NeuN^+^; ANOVA *F*_(2,43)_ = 20.62, *p* < 0.001 with TgN3^*WT*^< WT *p* < 0.001 and TgN3^*R169C*^< WT *p* < 0.001] and an increase in astrocytic differentiation [*F*_(2,43)_ = 12.01, *p* < 0.0001 with TgN3^*WT*^> WT *p* = 0.013 and TgN3^*R169C*^> WT *p* < 0.0001; Figure 3C,E,F]. While net gliogenesis was not altered [ANOVA *F*_(2,44)_ = 0.39 *p* = 0.67], net neurogenesis was reduced in both N3 and CADASIL transgenic animals [ANOVA *F*_(2,44)_ = 12.19, *p* < 0.0001 with TgN3^WT^ < WT *p* = 0.002 and TgN3^*R169C*^ > WT *p* = 0.0002; Figure 3D]. The morphology of BrdU^+^ new neurons and new astrocytes in both N3 and CADASIL transgenic mice appeared normal as shown in Figures 3E,F. For further confirmation of these results, we counted the number of calretinin^+^ cells in the SGZ of the DG as an independent marker for immature neurons in the adult dentate gyrus (Kempermann et al., 2004; Mattiesen et al., 2009; Knoth et al., 2010). A reduction in the number of calretinin^+^ cells was found [ANOVA *F*_(2,46)_ = 8.61 *p* = 0.0007 with TgN3^WT^< WT *p* = 0.018 and TgN3^*R169C*^ < WT *p* = 0.001; Figure 3A], and thus these results were in line with the BrdU data (representative images shown in Supplementary Figure 1).

In conclusion, N3 overexpression reduced survival and differentiation potential of precursor cells in adulthood with no significant additional effect of the CADASIL mutation. A correlation analysis was carried out (Supplementary Figure 2) to gain further information about a link between the mutation, the neurogenesis effect and the behavioral findings. In the TgN3^WT^ mice, a strong cluster of neurogenesis and gliogenesis parameters was found, which was weaker in WT and TgN3^*R169C*^ animals. Further, a positive correlation between water maze performance and adult neurogenesis was detected as described previously (Kempermann and Gage, 2002). Adult neurogenesis correlated well to the performance on the last day of acquisition (day 4) when the mice were most efficient in finding the hidden platform. CADASIL transgenic animals on the other hand did not show such strong positive correlations between adult neurogenesis and hippocampus-dependent learning, although they overexpressed N3 to the same extent. Thus, TgN3^*R169C*^ showed clusters of positive correlations for water maze performance throughout all trials, which indicated that these mice behaved more predictable and therefore, presumably, less flexible. This is corroborated by the finding that a correlation to neurogenesis could be detected only on the first day of reversal, when relearning is just starting and mice randomly search for the new platform.

In conclusion, the correlation analysis indicated that N3 is a regulator of adult neurogenesis and gliogenesis and might influence hippocampus-dependent learning in our animal model. The CADASIL mutation partly reverted the behavioral effects of N3 but not on adult neurogenesis.

### Deficits in Spatial Learning and Reduced Neurogenesis in N3 Transgenic Mice at 6 Months of Age

As first GOM accumulations were already present at 6 months of age (Figure 1), we also asked whether the described phenotypes were already present at this much younger age, possibly offering insight into the developmental process that leads to the described changes.

**FIGURE 4:**
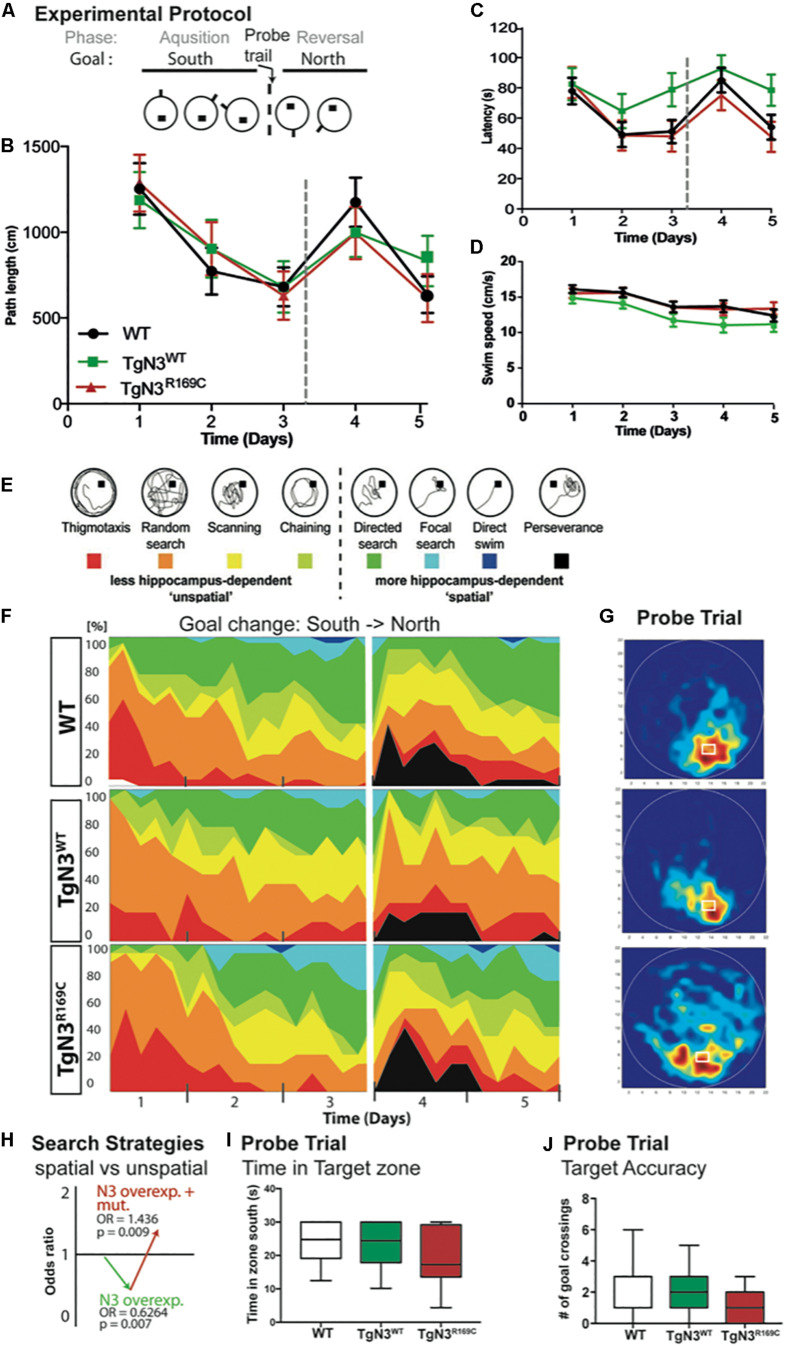
Morris water maze task in *Notch3* and CADASIL transgenic mice at 6 months of age. **(A)** The experimental protocol of the Morris water maze task containing 3 days of acquisition and 2 days of reversal. **(B)** Analysis of path length showed no deficits in performance. **(C)** Analysis of latency showed deficits, but since swim speed is reduced in TgN3^WT^ path length is a more reliable measure. **(D)** Swim speed of TgN3^WT^ mice is reduced compared to WT and TgN3^*R169C*^. **(E)** Classification of search strategies. **(F)** Contribution of the respective search strategies to group performance, color code as indicated in **(E)**. **(G)** Probe trial performance of the respective animal groups indicated by heat maps (only the first 30 s were plotted). Dark-red zones represent a 6-fold presence probability. **(H)** Graphic illustration of the induced change in the odds to choose a more hippocampus-dependent strategy, which is determined by logistic regression analysis based on classification shown in **(E)**. The values indicated that Notch3 overexpression leads to the use of less spatial cues and that the CADASIL mutation *per se* results in the use of more spatial cues, which brought TGN3^*R169C*^ mice back to WT levels. **(I)** Analysis of time spent in target zone during the first 30 s of probe trial as indicator of target accuracy. **(J)** Analysis of former goal crossings as a precise indicator of target accuracy in the probe trials. Although CADASIL transgenic mice showed less target accuracy of the previous goal location, no significant changes can be detected with the applied measures.

At 6 months of age, CADASIL and N3 transgenic mice on the B6 background learned the Morris water maze task quickly and did not show any deficits during acquisition. The analysis of latencies throughout the experiment, with 3 days acquisition and 2 days reversal (Figure 4A), showed no significant differences of N3 transgenic mice to find the hidden platform (Chi-Square = 5.2, *p* = 0.09; Figure 4C). Swim speed, however, differed significantly (Chi-Square = 8.4, *p* = 0.008; Figure 4D), as TgN3^WT^ mice were significantly slower compared to WT (*p* = 0.006). Consequently, the analysis of path length as a less confounded measure was used, but did not reveal a significant difference (Chi-Square = 0.77 *p* = 0.76; Figure 4B). Although CADASIL mice showed slight deficits in remembering the previous platform location as the heat maps of the probe trial illustrate (Figure 4G). Nevertheless, analysis of probe trial performance revealed no significant differences in target accuracy, which was assessed by the number of former goal crossings (Chi-Square = 0.625, *p* = 0.73; Figure 4J) and the time spent in the target zone [ANOVA *F*_(2,53)_ = 2.073, *p* = 0.135; Figure 4I]. A closer look into the learning strategies (Figure 4E) revealed that N3 overexpression led to a reduction of spatial, hippocampus-dependent strategies (Figure 4F). Analysis of the spatial versus non-spatial strategies by logistic regression revealed an OR = 0.62 (*p* = 0.007) for N3 overexpressing mice (Figure 4H). The CADASIL mutation on the other side resulted in an OR = 1.436 (*p* = 0.009), indicating that at this age CADASIL mice used more spatial cues as N3 transgenic controls.

**FIGURE 5:**
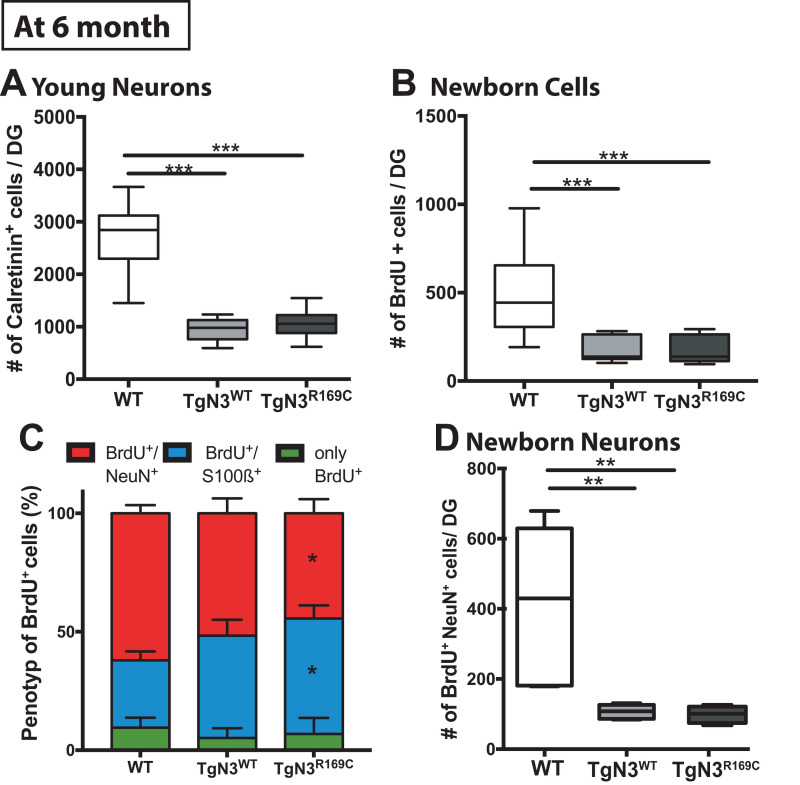
Reduced adult neurogenesis in *Notch3* and CADASIL transgenic mice at 6 months of age. **(A)** The number of calretinin^+^ cells, as a marker for young neurons, is reduced in the SGZ of the DG of Notch3 and CADASIL transgenic mice. **(B)** Analysis of adult neurogenesis by the number of BrdU^+^ cells 28 days post injection showed a reduction in Notch 3 and CADASIL transgenic animals. **(C)** Phenotyping of BrdU^+^ cells with neuronal marker (NeuN) and astrocyte marker (S100β) showed decreased neuronal differentiation and an increased astrocytic differentiation in CADASIL transgenic mice compared to WT, Data = Mean ± SEM. **(D)** Net neurogenesis, as calculated by number of BrdU^+^/NeuN^+^ cells, is reduced in Notch3 and CADASIL transgenic mice. *N* = 15 WT and 12 for TgN3WT and TgN3^*R169C*^ in all graphs. Asterisks indicate statistical significance (*p < 0.05, **p < 0.01, ***p < 0.005), DG, dentate gyrus; SGZ, subgranular zone.

As hippocampus-dependent learning seemed to be partially impaired in N3 transgenic mice at 6 months of age, we carried out a detailed analysis of precursor proliferation and differentiation in the adult hippocampus. As expected from our study on the FVB/N background (Ehret et al., 2015), histological analysis revealed that adult neurogenesis, in particular precursor proliferation and differentiation, was impaired at 6 months in N3 and CADASIL transgenic mice to the same extent. Hence, also the number of young neurons, as analyzed by calretinin immune labeling (Supplementary Figure 1), was significantly reduced in N3 and CADASIL transgenic mice [ANOVA *F*_(2,35)_ = 85, *p* < 0.0001 with TgN3^WT^ < WT, *p* < 0.0001 and TgN3^*R169C*^ < WT *p* < 0.0001; Figure 5A]. Similar results were obtained by the analysis of BrdU^+^ cells 28 days post injection [ANOVA *F*_(2,35)_ = 19.56, *p* < 0.0001 with TgN3^*WT*^< WT, *p* < 0.0001 and TgN3^*R169C*^< WT, *p* < 0.0001; Figure 5B; representative images Supplementary Figure 1]. Phenotyping of BrdU^+^ cells revealed a reduction in neuronal differentiation (as measured by % of BrdU^+^/NeuN^+^ co-labeling) in CADASIL transgenic mice in comparison to WT mice [ANOVA *F*_(2,25)_ = 3.41, *p* = 0.0049 with TgN3^*R169C*^ < WT, *p* = 0.047; Figure 5C]. Further, an increase in astrocytic differentiation (measured by % of BrdU^+^/S100β^+^ co-labeling) in CADASIL transgenic mice in comparison to WT mice [ANOVA *F*_(2,25)_ = 4.62, *p* = 0.019; TgN3^*R169C*^> WT *p* = 0.025] was observed. As a result, net neurogenesis was reduced in N3 and CADASIL transgenic mice [ANOVA *F*_(2,13)_ = 10, *p* = 0.002 with TgN3^*WT*^< WT, *p* = 0.004 and TgN3^*R169C*^< WT, *p* = 0.007; Figure 5D]. Nevertheless, net gliogenesis was not altered [ANOVA *F*_(2,13)_ = 1.2, *p* = 0.32]. These results indicate that in N3 overexpressing mice, adult neurogenesis is already impaired at an early age, which is in line with the observed albeit mild deficits in hippocampus-dependent spatial search strategies in the Morris water maze task. The CADASIL mutation in contrast, did not lead to an additional impairment in precursor proliferation or differentiation in this transgenic mouse model on C57BL/6J background.

### Attempt to Study Adult Neurogenesis in CADASIL Patient Samples

**FIGURE 6:**
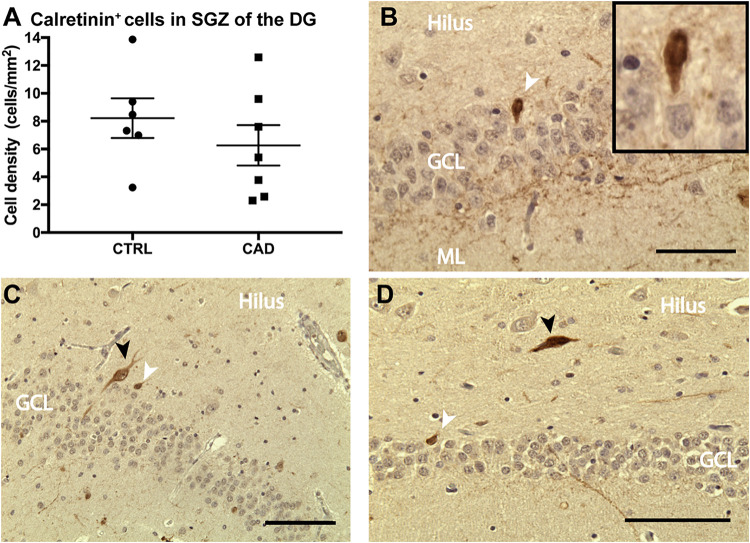
Calretinin expression in the dentate gyrus of CADASIL post-mortem samples. **(A)** Number of calretinin^+^ new born neurons per area of the granular cell layer (GCL) of control (CTRL) and CADASIL (CAD) patient samples. Cell density was averaged over 4–6 sections per patient. **(B–D)** Representative micrograph of calretinin^+^ new born neurons (white arrow) and larger Interneurons (black arrow), which are mainly found the hilus of human DG from CADASIL **(B,D)** and Control patients **(C)**. Tissue was counter stained by haematoxylin. ML, molecular layer. Data = Mean ± SEM, *N* = 6–7, Scale bar 50 μm.

Based on our current and previous observation (Ehret et al., 2015) of a potential direct regulatory influence of N3 on adult hippocampal neurogenesis and the loss-of-function deficits seen in CADASIL transgenic mice, we intended to analyze adult neurogenesis also in patient samples. We were aware of the fact that there are substantial challenges to assessing adult neurogenesis in human specimens (Kempermann et al., 2018; Moreno-Jimenez et al., 2019). Histological analysis on hippocampal samples collected from the CADASIL brain bank (Leiden, Netherlands) showed calretinin-positive cells in the dentate gyrus (Figure 6B) of both patient and control specimens, indicative of adult hippocampal neurogenesis (Brandt et al., 2003; Mattiesen et al., 2009; Knoth et al., 2010; Boldrini et al., 2018). Interneurons were distinguished from immature granular cells based on size and location (shown in Figures 6C,D). Additional neurogenic markers were tested but did not produce convincing results in these specimens (see Supplementary Table B). All samples were checked for confounding effects of age, sex and post-mortem interval, when the areal density of calretinin-positive cells was assessed (Supplementary Figure 3). Altough post-mortem delay did not negatively influence density, no statistical differences in density of calretinin-positive cells were found (Figure 6A). Given the rareness of this disease (and related tissue samples), it was unfortunate that no stereological quantification was possible and the results remain unconclusive with respect to a potential effect of CADASIL on adult neurogenesis.

## Discussion

The present study aimed at further elucidating the potential involvement of the hippocampus and failing adult neurogenesis in the manifestation of CADASIL. We had previously shown that N3 plays an essential role in adult neurogenesis, controlling aspects of precursor proliferation, activation and differentiation (Ehret et al., 2015). This is of particular relevance for CADASIL, since the functional consequences of the several established CADASIL mutations, including the R169C mutation investigated here, have still no yet been comprehensively described. The current study identified deficits in hippocampus-dependent spatial memory in CADASIL transgenic mice. The observed behavioral phenotype was mild and could not be explained by N3-dependent effects on adult neurogenesis, because the mutation did not affect the neurogenesis phenotype beyond the N3 effect.

At the same time we confirmed that a regulatory influence of N3 appears to be critical for a variety of neural stem and precursor cells (Kawai et al., 2017; Than-Trong et al., 2018). The differential activation and differentiation of stem and precursor cells might lead to a deficient use of spatial search strategies. Previous studies had shown that, if adult neurogenesis was abolished, as for example in Cyclin D2 knockout mice (Garthe et al., 2014), after irradiation (Snyder et al., 2005), or after treatment with a cytostatic agent (Garthe et al., 2009; Goodman et al., 2010), flexible re-learning was impaired. In the present study we could show that with increasing age especially this reversal learning is impaired in R169C CADASIL transgenic mice. So far, only one other CADASIL study had looked into spatial learning, but re-learning had not been assessed (Liu et al., 2015). The limitation of the model lies on the fact that it relies on N3 overexpression. Some of the effects of the mutation (on the ratio of spatial vs. unspatial strategies but not on perseverance) unveiled a loss of function that reverted or counteracted the pure N3 effects.

The used animal model is based on a strong (4-fold overexpression) of N3 with an endogenous-like expression pattern due to a PAC-based transgenesis approach (Joutel et al., 2010). Both transgenic lines express rat N3 at similar levels in addition to the endogenous mouse N3 protein (Supplementary Figure 4). Since the overexpression *per se* has such a strong effect on stem and progenitor cells, the additional functional deficits observed through the mutation are rather small and thus hard to distinguish from the overexpression phenotype. Nevertheless only models with at least 2–4 fold overexpression have been able to represent the spectrum of disorders observed in CADASIL patients like the occurrence of GOMs, white matter brain lesions and vascular deficits (Joutel et al., 2010). In comparison to the previous evaluation of this model on the FVB/N background, fewer deficits could be detected in neurogenesis at the early stage of 6 months. Our studies thus add to the body of literature that describes how strongly the effect of a mutation depends on the genetic background.

Generally, in dementias, such as in Alzheimer’s disease, re-learning and recall are prominently affected (Karantzoulis and Galvin, 2011; Tort-Merino et al., 2017). In CADASIL patients this has not yet been specifically analyzed and only deficits in working memory, short-term memory, recall of verbal memory as well as executive and organizational functions have been studied (Taillia et al., 1998; Amberla et al., 2004; Epelbaum et al., 2011). Impaired adult neurogenesis does not affect spatial learning *per se*; it is relevant for specific aspects like up-dating of a previously formed cognitive map (Zhang et al., 2008; Garthe et al., 2014) and behavioral pattern separation (Clelland et al., 2009). There is theoretical and experimental evidence to support that adult neurogenesis levels do not correlate with probe trial performance but with water maze acquisition (Kempermann and Gage, 2002). Our data further support this view since adult neurogenesis (BrdU^+^/NeuN^+^) correlated to water maze performance (Day 2 and 4) for WT and TgN3^WT^ mice (Supplementary Figure 2). However, no correlation between adult neurogenesis and the acquisition phase of Morris water maze was found in CADASIL transgenic mice; only a correlation to the reversal phase and perseverance was observed. This indicates that aged CADASIL transgenic mice partially lack the flexibility of relearning a new spatial position. The use of spatial search strategies was impaired in N3 transgenic mice and almost back to WT levels in CADASIL transgenic mice, which partially supports a partial loss of function phenotype as found previously for this CADASIL mutation (Ehret et al., 2015). N3 overexpression on the side leads to predominantly less hippocampus-dependent strategies (Figure 2H) and perseverance after platform reversal.

Thus, these results confirm the critical involvement of N3 in precursor cell proliferation, activation and differentiation (Ehret et al., 2015; Kawai et al., 2017; Than-Trong et al., 2018), which might have direct functional consequences for the flexible integration of new information into previously established contexts (Garthe et al., 2009; Garthe and Kempermann, 2013). On the other hand, these results show that the impairment seen in CADASIL transgenic mice due to the mutation itself seems to be an additional component diminishing only certain aspects of plasticity and thus influencing the up-dating of the existing allocentric map, but do not *per se* lead to a loss of function phenotype. GOM accumulations and other subtle aspects of the pathology might additionally impair hippocampal function. Taken together, a complex relationship of vascular and neurogenic origin might influence hippocampus-dependent function in CADASIL, specifically with respect to the R169C mutation.

Since we know the limitation of the available murine CADASIL models, we also intended to analyze adult neurogenesis in human tissue sections. We had the opportunity to undertake the present study with rare CADASIL tissue samples from Leiden University and tested several histological markers (DCX, PCNA, Prox1, Sox2) and protocols (for details see Supplementary Table B) based on previously described markers (Knoth et al., 2010). Of these, only Calretinin immune reactivity could be consistently established. Further the structural diversity in the human hippocampal samples made stereological investigations impossible so that no strong quantitative data could be obtained. This limitation was further aggravated by the fact that cell counts needed to be normalized to area of the granular cell layer (Moreno-Jimenez et al., 2019), a common approach to compensate for the comprehensive stereotaxic analysis that would otherwise be desirable but relies on homogenous tissue samples or very high N. Nevertheless, to grossly evaluate potential region-specific differences, we subdivided the hippocampus into head, body and tail structures (Duvernoy and Vannson, 1988). Of these, the head region seemed to show some prominent changes in cell density (Supplementary Figure 5). Regional specific differences have not been evaluated by MRI in CADASIL patients so far, but the hippocampal head is sensitively affected in Alzheimer’s disease and mild cognitive impairment (Wang et al., 2003; Feng et al., 2018), schizophrenia (O’Driscoll et al., 2001), epilepsy (Bernasconi et al., 2003), and traumatic brain injury (Ariza et al., 2006). Regional differences, if confirmed, would therefore be in line with other studies. Other than that, our study unfortunately remained inconclusive with respect to adult neurogenesis in patient samples.

We present the data nevertheless, because omitting the description of the unsuccessful attempt would create a publication bias in that positive findings are generally more likely to be published than negative or ambiguous results. Data on adult hippocampal neurogenesis in humans are scarce and problematic to obtain, partly because of the limited availability of specimen to be studied. This applies particularly to disease cases, such as CADASIL. With calretinin we could only assess one, obviously robust, marker, which by itself could not settle the case. But it is important that attempts are published and the difficulties and challenges are made public, because of the high expectations on studies on adult neurogenesis in disease contexts to include human data.

Independent of this question, our study provides evidence for an involvement of N3 in structural and functional plasticity of the hippocampus by controlling aspects of cell proliferation, activation and differentiation, which has functional consequences for spatial memory. In addition, the CADASIL mutation seems to partially impair N3 function affecting spatial memory function in the Morris water maze task.

## Data Availability Statement

The raw data supporting the conclusions of this article will be made available by the authors, without undue reservation.

## Ethics Statement

The animal study was reviewed and approved by the Landesdirektion Sachsen, Dresden, Germany. For the provided human data, all participants gave informed consent that their samples could be used for research and publication.

## Author Contributions

FE and GK planned the study and wrote the manuscript. FE, RMT, M-TN, BH, and DL performed the experiments. FE analyzed the data. GK supervised the entire project and provided financial support. All authors contributed to the article and approved the submitted version.

## Conflict of Interest

The authors declare that the research was conducted in the absence of any commercial or financial relationships that could be construed as a potential conflict of interest.

## Abbreviations

BrdU: Bromodeoxyuridine
CADASIL: Cerebral autosomal dominant arteriopathy with subcortical infarcts and leukoencephalopathy
DG: dentate gyrus
GFAP: glial fibrillary acidic protein
GOM: granular osmiophilic material
PFA: paraformaldehyde
N3: Notch3
RT: room temperature
TBS: tris-buffered saline
TgN3^WT^: transgenic mice overexpressing rat wild type Notch3
TgN3^*R169C*^: transgenic mice overexpressing rat CADASIL mutant Notch3
SGZ: subgranular zone.
