# Supplementary material for: Notch3-Dependent Effects on Adult Neurogenesis and Hippocampus-Dependent Learning in a Modified Transgenic Model of CADASIL
Source: Front Aging Neurosci. 2021 May 21;13:617733. doi: 10.3389/fnagi.2021.617733 (PMC8177050; doi:10.3389/fnagi.2021.617733)
Supplement: Supplementary file 1 [file Table_1.docx]

Supplementary Material

**Table A**, Human sample characteristics. Provided from brain bank at Leiden University.

| Patient group | Age | Gender | Post mortem delay (h) |
| --- | --- | --- | --- |
| CAD | 61 | M | 8 |
| CAD | 59 | F | 19 |
| CAD | 58 | F | 17 |
| CAD | 57 | M | 3.5 |
| CAD | 69 | M | 3 |
| CAD | 55 | M | 3.5 |
| CAD | 53 | M | unknown |
| CTR | 68 | M | 29 |
| CTR | 48 | M | 17 |
| CTR | 51 | F | 12 |
| CTR | 69 | M | Unknown |
| CTR | 61 | F | 11 |
| CTR | 54 | M | 10 |

**Table B**, Tested antibodies and Epitope retrieval protocols.

| Antigen | Antibody  (Lot No.) | Dilution factor | Company | Antigen unmasking procedure | DAB detection |
| --- | --- | --- | --- | --- | --- |
| Calretinin | CR7697 Lot.1893-0114 | 2000  500 - 2000 | Swant  Swant | 1 mM ETDA (pH 8)  10 mM citric acid (pH 6) | Worked  Weak |
| DCX | ab18723  AB2253 | 250 - 1000  500 - 5000 | Abcam  Millipore | 1 mM ETDA (pH 8)/ 10 mM citric acid (pH 6)  1 mM ETDA (pH 8)/ 10 mM citric acid (pH 6) | Neg.  Neg.  Neg.  Neg. |
| PCNA | ab18197 | 200 - 1000 | abcam | 1 mM ETDA (pH 8)  10 mM citric acid (pH 6) | Neg.  Neg. |
| Prox1 | AB5475 | 200 - 800 | Cheminon | 1 mM ETDA (pH 8)  10 mM citric acid (pH 6) | Neg.  Neg. |
| Sox2 | sc17320 | 100 - 500  100 - 500 | Santa Cruz | 1 mM ETDA (pH 8)  10 mM citric acid (pH 6) | Neg.  Neg. |

Neg. means that no detachable staining was observed, which might be due to over-fixation of the material. DAB, diaminobenzidine detection method

**
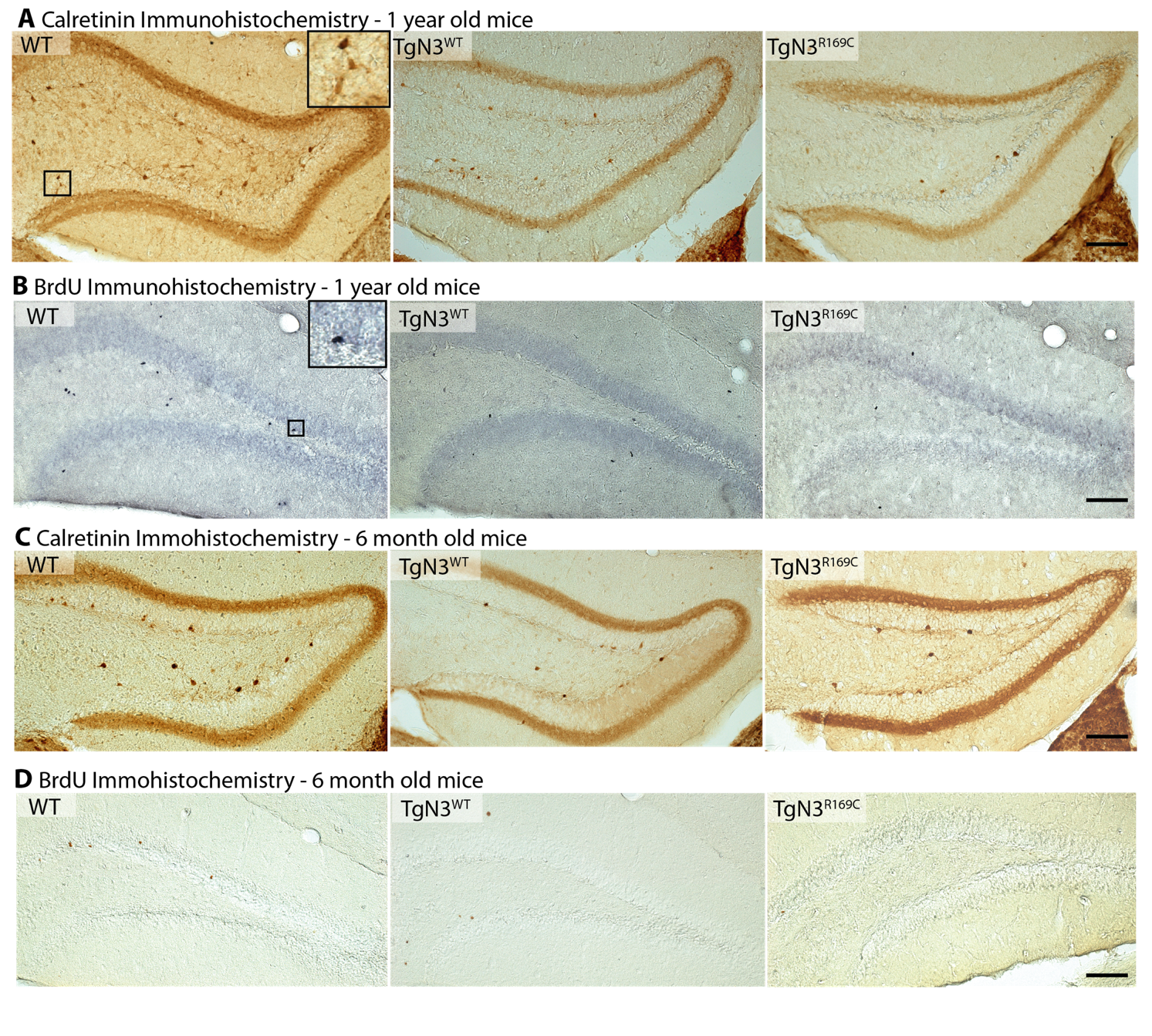
**

**Figure S1.** Immunohistochemistry analysis of Calretinin and BrdU in WT, N3 and CADASIL transgenic animals

A, Representative images of Calretinin^+^ DAB cells in the DG of 1 year old mice. In the Inlet a higher magnification of two Calretinin+ cell can be seen. Only cell in the subgranular zone and molecular layer of the DG were quantified. B, Representative images of BrdU^+^ DAB cells in the DG of 1 year old mice. C, Representative images of Calretinin^+^ DAB cells in the DG of 6 month old mice. D, Representative images of BrdU^+^ DAB cells in the DG of 6 month old mice.

All images are taken with the same magnification Scale bar = 100µm.


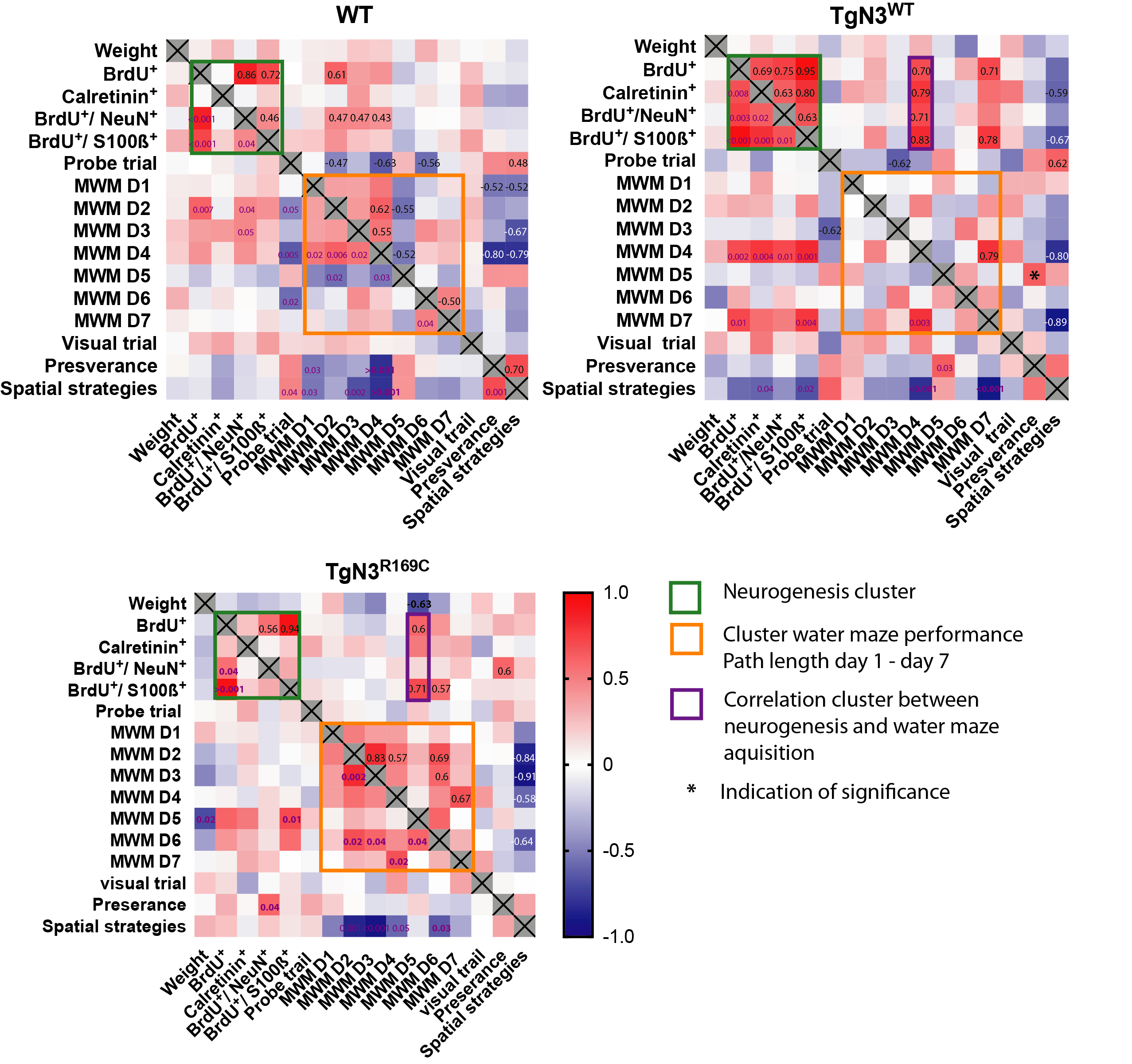


**Figure S2**. Correlation between adult neurogenesis and spatial learning in the Morris water maze (MWM) in N3 and CADASIL transgenic mice at 12 months of age. Neurogenesis parameter like number of cells per mouse which were calretinin^+^, BrdU^+^, BrdU^+^/NeuN^+^ or BrdU^+^/S100β^+^ are correlated to water maze parameters like length of the probe trial, day 1- day 7, visual trial and the summed-up use of spatial strategies as well as perseverance. Positive and negative correlations are shows as indicated by the legend. Spearmanns R was used for calculation. N = 19 for WT and 13 for both TgN3 lines. For each matrix, significant interactions are indicated by placing correlation strength on upper right half and p-values in purple on the lower left half. At this sample size a Spearmanns R correlation of greater than 0.47 and lower then -0.47 are seen as relevant interaction.


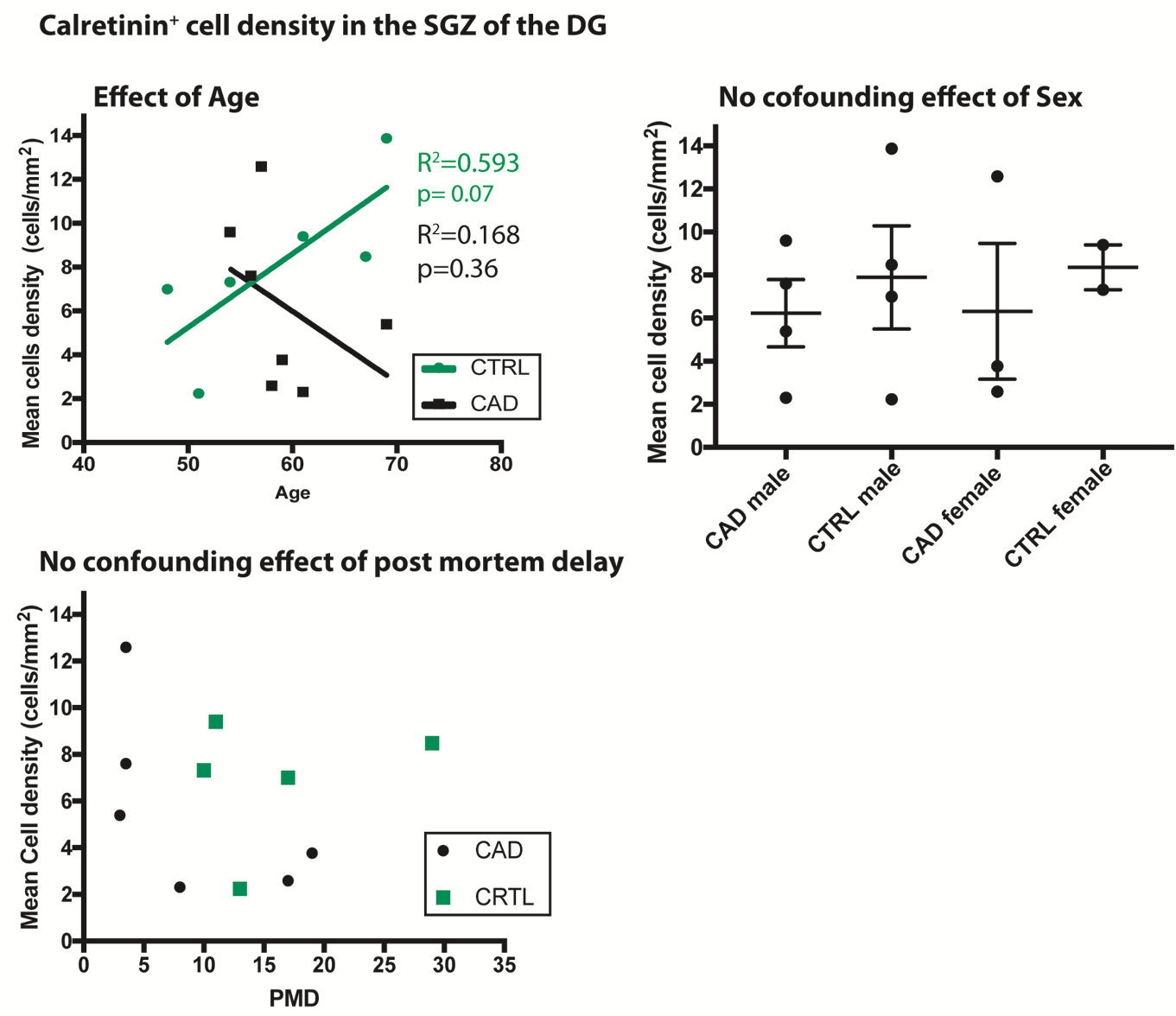


**Figure S3**. Influence of age sex and post mortem delay (PMD) on cell density of calretinin^+^ cells in the human DG. Each point represents one averaged patient sample with 6 measurements per patient. The effect of age was analysed by linear regression and showed that control samples (CTRL) do not decrease in cell density with age whereas CADASIL samples (CAD) show trends towards reduced cell density with age. The effect of sex (right panel) is shown as mean +/- SEM.


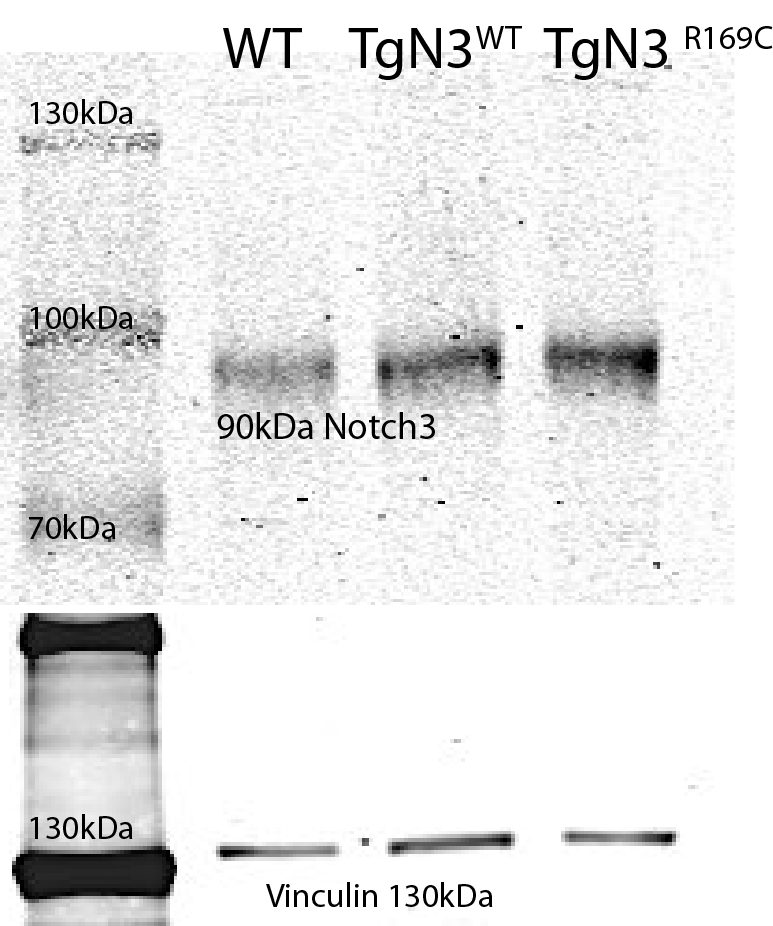


**Figure S4.** N3 protein expression in the dentate gyrus of WT, N3 and CADASIL transgenic mice.

N3 intracellular domain can be seen at 90kDa in all 3 mouse lines. Expression of N3 in WT mice was weaker but similar between the two transgenic lines as quantified by image analysis through Fiji. We detected a 2.9- to 3.2 -fold overexpression in both N3 transgenic lines compared to WT mice. As loading control Vinculin was used.


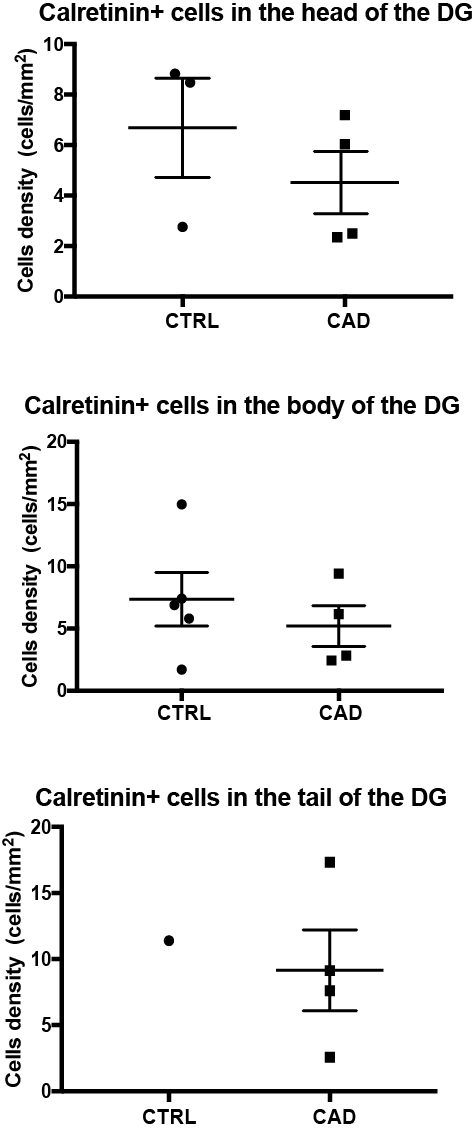


**Figure S5**, Calretinin^+^ cells in the human head, body and tail region of dentate gyrus analysed from post mortem tissue of 7 CADASIL and 6 control patients.
